# Benchmark Dose of Melamine Exposure for a Renal Injury Marker Mediated by Oxidative Stress: Examples in Patients with Urolithiasis and Occupational Workers

**DOI:** 10.3390/toxics12080584

**Published:** 2024-08-11

**Authors:** Chu-Chih Chen, Chia-Chu Liu, Yin-Han Wang, Chia-Fang Wu, Yi-Chun Tsai, Sih-Syuan Li, Tusty-Jiuan Hsieh, Ming-Tsang Wu

**Affiliations:** 1 Institute of Population Health Sciences, National Health Research Institutes, 35 Keyan Road, Miaoli 350401, Taiwan; yhwang27@gmail.com; 2 Research Center for Precision Environmental Medicine, Kaohsiung Medical University, Room 721, CS Research Building, 100 Shih-Chuan 1st Road, Kaohsiung 807378, Taiwan; ccliu0204@gmail.com (C.-C.L.); chiafangwu27@gmail.com (C.-F.W.); lidam65@yahoo.com.tw (Y.-C.T.); ss770220@gmail.com (S.-S.L.); hsiehjun@kmu.edu.tw (T.-J.H.); 3 Department of Urology, Kaohsiung Medical University Hospital, Kaohsiung Medical University, Kaohsiung 807378, Taiwan; 4 Department of Urology, Pingtung Hospital, Ministry of Health and Welfare, Pingtung City 90054, Taiwan; 5 International Master Program of Translational Medicine, National United University, Miaoli 360301, Taiwan; 6 Division of Nephrology, Department of Internal Medicine, Kaohsiung Medical University Hospital, Kaohsiung Medical University, Kaohsiung 807378, Taiwan; 7 Graduate Institute of Medicine, College of Medicine, Kaohsiung Medical University, Kaohsiung 807378, Taiwan; 8 Department of Marine Biotechnology and Resources, College of Marine Sciences, National Sun Yat-sen University, Kaohsiung 804201, Taiwan; 9 Department of Public Health, Kaohsiung Medical University, Kaohsiung 807378, Taiwan; 10 Department of Family Medicine, Kaohsiung Medical University Hospital, Kaohsiung Medical University, Kaohsiung 807378, Taiwan; 11 Ph.D. Program in Environmental and Occupational Medicine, Kaohsiung Medical University, Kaohsiung 807378, Taiwan

**Keywords:** benchmark dose, direct effect, indirect effect, Markov chain Monte Carlo, mediation, tolerable daily intake

## Abstract

Establishing a safe exposure level from epidemiological studies while providing direct hazard characterization in humans often faces uncertainty in causality, especially cross-sectional data. With advances in molecular epidemiology, it is reasonable to integrate identified intermediate biomarkers into health risk assessment. In this study, by considering the mediation of the oxidative stress marker malondialdehyde (MDA), we explored the exposure threshold of melamine on the early renal injury marker N-acetyl-β-D glucosaminidase (NAG). The benchmark dose (BMD) was derived from model averaging of the composite direct effect of melamine exposure and the indirect effect through the mediation of MDA on NAG levels. As illustrative examples, we analyzed 309 adult patients with calcium urolithiasis and 80 occupational workers for the corresponding exposure thresholds. The derived threshold was subpopulation-dependent, with the one-sided lower bound BMDL_10_ for the patients with urolithiasis with (without) the mediator MDA for the patients with kidney stones and the occupational workers being 0.88 (0.96) μg/kg_bw/day and 22.82 (18.09) μg/kg_bw/day, respectively. The derived threshold levels, considering the oxidative stress marker MDA, were consistent with those without adjusting for the mediation effect. However, the study outcomes were further supported by the suggested mechanism pathway. The threshold for the patients with urolithiasis was up to two orders lower than the current tolerable daily intake level of 200 μg/kg_bw/day recommended by the WHO (EFSA).

## 1. Introduction

Melamine, a chemical that is produced in high volume and used in the manufacture of laminates, plastics, glues, adhesives, coatings, and derivatives used in flame retardants and insulation, are ubiquitously present in the general environment [1,2]. Being widely used as a substitute for porcelain, melamine-made tableware and food utensils have been found to be a major exposure source in the Taiwanese population [3,4,5].

Because of the high content of nitrogen, melamine was deliberately added to animal feeds to give the false impression of increased protein levels. Previous food scandals involving melamine led to thousands of pet animal deaths in 2007 in the United States and more than 50,000 cases of nephrolithiasis in children in China [2,6,7]. In addition to acute health effects due to melamine adulteration, environmental low-concentration melamine exposure has been shown to be associated with elevated N-acetyl-beta-D-glucosaminidase (NAG), an early renal injury marker, in patients with urolithiasis [8]. Long-term follow-up studies have also found associations with adverse kidney outcomes in patients with diabetes mellitus and kidney function deterioration in patients with early chronic kidney disease (CKD) [9,10]. In addition, co-exposure to melamine and di-(2-ethylhexyl) phthalate (DEHP) was found to be associated with elevated NAG in the third trimester of women who are pregnant [11]. Moreover, Liu et al. showed that the oxidative stress marker malondialdehyde (MDA) plays a mediating role between melamine levels in urine and levels of NAG, an early renal injury marker [12]. The indirect effect (IE) of the total effect of melamine exposure on NAG mediated by MDA accounted for 36% to 53%. Thus, the pathway between low-dose melamine exposure and the risk of early kidney damage in humans was established.

Given a benchmark response (BMR) of 0.10, the 95% lower bound of the benchmark dose (BMD), BMDL_10_ (regarded as the exposure threshold), of melamine for patients with urolithiasis and patients with early-stage CKD was shown to be 4.89 and 0.74–2.03 μg/kg bw/day, respectively [13,14]. In addition, the BMDL_10_ level of melamine exposure on elevated NAG levels in women who are pregnant was 2.67 μg/kg_bw/day [15]. All the derived exposure thresholds for melamine based on clinical observations and epidemiological studies were one to two orders lower than the current WHO recommended tolerable daily intake (TDI) of 200 μg/kg_bw/day and the US FDA recommended reference dose of 63 μg/kg_bw/day, both of which are based on the same animal study data.

While the derived melamine exposure thresholds from different epidemiological datasets were consistent, the study outcomes may suffer from a lack of evidence of causality and other uncertainties. In this study, as an alternative approach, we integrated the suggested mechanism pathway in deriving the BMDL_10_ level of melamine so that causal inference may be strengthened and more supportive. Following the study outcomes of Liu et al. [12], we reanalyzed the patients with urolithiasis from the study by Wang et al. [14] by considering the composite direct effect (DE) of melamine exposure and the IE of mediation of the oxidative stress marker MDA for BMDL_10_. To further support the methodology, a second dataset of 80 occupational workers was analyzed using the same proposed procedure.

## 2. Materials and Methods

### 2.1. Study Subjects

Two datasets from previous epidemiological studies were analyzed for the melamine exposure threshold for NAG with a mediating effect on the oxidative stress marker MDA [8,12]. The first was a clinical cross-sectional study of 309 patients with urolithiasis. The second was an occupational exposure study with 38 workers from a melamine tableware factory and 42 workers from a large steel company as the control group.

#### 2.1.1. Patients with Calcium Urolithiasis

A total of 309 patients aged ≥ 20 years diagnosed with upper urinary tract urolithiasis, recruited from three Kaohsiung Medical University-affiliated hospitals in southern Taiwan between 2013 and 2015, were analyzed [8,12]. All patients provided kidney stone specimens that were confirmed by infrared spectroscopy to have calcium components. One-spot morning first-void urine samples were collected during admission. To collect detailed demographic data and medical history, the patients were interviewed by trained researchers using a structured questionnaire. Subjects who had regularly consumed any alcohol ≥ once, smoked ≥ 10 cigarettes, or had chewed ≥ 7 betel nuts per week for more than six months were defined as alcohol drinkers, cigarette smokers, or betel nut chewers, respectively.

#### 2.1.2. Occupational Workers

We recruited 38 melamine-exposed workers (the melamine-exposed group) from a melamine tableware factory and another 42 workers (the nonexposed group) from a large steel company in 2012. The eligible melamine-exposed workers studied here had worked in the melamine tableware factory for one year or more. According to work activities, the work sites were categorized into manufacturing, grinding, packing, and administration areas [12,16]. The workers in the nonexposed group were office staff from one large steel company and had no current or past exposure to melamine or other chemicals known to cause kidney injury [16].

To collect one-spot urine samples for measurements of melamine and other markers of oxidative stress and kidney function, we used two approaches: series collection and one-time collection [16]. For the series collection of one-spot urine samples, we collected one-spot urine samples in the preshift (early morning before leaving for work each day) and postshift (in the evening after work each day) for five consecutive weekdays from Monday to Friday and the following early morning of Saturday, Sunday, and the following Monday in the melamine-exposed group only. Thus, each melamine worker had a total of 13 one-spot urine samples [12,16].

For the one-time collection of one-spot urine samples, all workers from both the melamine-exposed group and the non-exposed group were first physically examined by occupational physicians, and then another one-spot urine sample was collected on the morning of the fifth working day (Friday) [16]. The study was approved by the Institutional Review Board of Kaohsiung Medical University Hospital (KMUH) and the Research Ethics Committee of the National Health Research Institutes (NHRI).

### 2.2. Measurements of Melamine and Oxidative Stress and Renal Injury Biomarkers in Urine

Melamine concentration in collected urine samples was measured using the liquid chromatography/tandem mass spectrometry method (LC–MS/MS) (API4000Q, Applied Biosystems/MDS SCIEX, Concord, ON, Canada), as described in previous studies [8,12,16]. Measurements below the limit of detection (LOD) of melamine (0.4 ng/mL) were recorded as half of the LOD. The urinary oxidative stress biomarker MDA was measured using high-performance liquid chromatography with fluorescence (HPLC-FL) detection with a reversed-phase column (Luna C18, 250 × 4.6 nm) [17]. The renal injury marker NAG was measured using an NAG assay kit from Diazyme (Diazyme Laboratories, Poway, CA) and was corrected by creatinine as U/mmol creatinine [11]. Urinary creatinine was assessed by spectrophotometry (U-2000: Hitachi, Tokyo, Japan) set at a wavelength of 520 nm to measure the creatinine-picrate reaction.

### 2.3. Estimation of Participant’s Average Daily Intake of Melamine

The estimated daily intake (EDI) of melamine adjusted by creatinine of the participants based on their urinary melamine levels is as follows:

(1) $$ED\mathrm{I\_c}r\;\left(\mu g/k\mathrm{g\_b}w/day\right)=\frac{M_{Cr}\left(\mu g/gCr\right)\times CE\left(gCr/kg/day\right)}{F_{UE}},$$

where $M_{Cr}$ is the creatinine-adjusted urinary melamine concentration, *CE* is the daily creatinine excretion rate, 0.023 g/kg_bw/day for men and 0.018 g/kg_bw/day for women [18], and $F_{UE}$ is the fraction of excreted melamine in urine relative to the total exposure. An excretion fraction of *F_UE_* = 0.26 was adopted in this study following pharmacokinetic study outcomes in rhesus monkeys [19] and other studies [13,14,15]. An $F_{UE}$ of 0.90 was also calculated as a comparison, which follows previous toxicokinetic studies in rats [20,21].

In addition, the participants’ EDI by covariate-adjusted creatinine standardization was estimated as follows:

(2) $$\mathrm{EDI\_cacs}\;(\mu g/\mathrm{kg\_bw}/\mathrm{day})=\frac{M_{V}\;(\mu g/L)\;\times \;U_{V}\;\left(L/\mathrm{day}\right)}{\mathrm{BW}\;(\mathrm{kg})\;\times \;\left(C_{r}/{\hat{C}}_{r}\right)\;\times \;F_{\mathrm{UE}}},$$

where $c_{r}$ and ${\hat{c}}_{r}$ are the observed and covariate-adjusted creatinine levels, respectively, and $U_{V}$, the daily voided urine volume, was simulated from a lognormal distribution $logN\left(0.42,\;0.38^{2}\right)$ with a mean of 1.63 L/day for both men and women [22]. The covariables adjusted in the (log-transformed) multivariate regression model for ${\hat{c}}_{r}$ included age, sex, and BMI, variables that are known to directly and chronically affect urine dilution [20,23].

### 2.4. Associations between Melamine Exposure and Participant’s Levels of NAG and MDA

Mediation analysis may be used to identify the pathway or mechanism of a potential mediator between exposure and an outcome and provide causal inference. Moreover, the relative magnitude of the attribution of the exposure through the mediator to the health outcome may be assessed through a set of regression models for the associations between exposure and outcome, exposure and mediator, and exposure and outcome in the presence of the mediator. In this subsection, we incorporated the mechanistic pathway of the oxidative stress marker MDA into the association between environmental exposure to melamine and the early renal injury marker NAG. First, a regression model for the total effect of melamine exposure on NAG was established. Second, a regression for the association between melamine exposure and the oxidative stress marker MDA was performed. Third, a regression model for the association between NAG and melamine exposure, together with the mediator MDA in the regression, was established. In this way, the direct effect (DE) of melamine exposure on NAG was the regression coefficient in the third model, whereas the indirect effect (IE) was the difference between the coefficient of melamine exposure in the first model and that in the third model [24]. Alternatively, the IE of melamine in NAG may be interpreted as the composite effect of melamine exposure on MDA in the second model and the effect of MDA on NAG in the third model [24].

Because the distributions of the participants’ NAG and MDA measurements and the EDI of melamine exposure were highly skewed, we log-transformed the NAG, MDA, and EDI of melamine exposure before regression. Specifically, we assumed that the association between the *i*th participant’s EDI of melamine exposure and his or her renal injury marker NAG level follows the statistical model, written as follows:

(3) $$log\left({NAG}_{i}\right)=\beta_{0}+\beta_{1}g\left(x_{i}\right)+\gamma_{1}^{\prime }Z_{i}+\varepsilon_{0,i},$$

where $g\left(.\right)$ is a monotone increasing function, $x_{i}=log\left({EDI}_{i}+1\right)$, $Z_{i}$ is the vector of the participant’s covariates, $\gamma_{1}^{\prime }$ is the transpose of the vector of regression coefficients, and $\varepsilon_{i}$ is distributed as $N\left(0,\;\sigma_{0}^{2}\right)$. Similarly, the association between the *i*th participant’s EDI of melamine and his or her oxidative stress MDA level is

(4) $$log\left({MDA}_{i}\right)=\alpha_{0}+\alpha_{1}x_{i}+\gamma_{2}^{\prime }Z_{i}+e_{i},$$

For the melamine workers, the association between the repeated measurements of the oxidative stress level MDA and their estimated daily EDIs of melamine is calculed as follows:

(5) $$log\left({MDA}_{ij}\right)=\alpha_{0}+a_{i}+\alpha_{1}x_{ij}+\gamma_{2}^{\prime }Z_{i}+e_{ij},$$

where *MDA_ij_* is the *j*th repeated measurement of the MDA level, $a_{i}$ is the random effect of the *i*th participant assumed to be distributed as $N\left(0,\;\sigma_{a}^{2}\right)$, and both $e_{i}$ and $e_{ij}$ are assumed to be distributed as $N\left(0,\;\sigma_{m}^{2}\right)$. The association between the *i*th participant’s EDI of melamine exposure and his or her renal injury marker NAG level mediated by MDA is assumed to be calculated as follows:

(6) $$log\left({NAG}_{i}\right)=\theta_{0}+\theta_{1}g\left(x_{i}\right)+\theta_{2}log\left({MDA}_{i}\right)+\gamma_{3}^{\prime }Z_{i}+\varepsilon_{1,i},$$

where $\varepsilon_{1,i}$ is assumed to be distributed as $N\left(0,\;\sigma_{1}^{2}\right)$. For simplicity, the functional form for the mediator MDA in Equation (6) was assumed to be logarithmically transformed. Given Equations (3)–(6), under the linear association setting ($g\left(x\right)=x$), the total effect of melamine exposure on NAG was the coefficient $\beta_{1}$ (Equation (3)). The DE was the coefficient $\theta_{1}$, and the IE was $\beta_{1}-\theta_{1}$ (Equation (6)) [24].

The covariables adjusted in the models (Equations (3)–(6)) were all the same, which included age, sex, body mass index (BMI), and smoking status. The covariables adjusted in the model for the occupational workers and the patients with kidney stones were employer and index of severity, respectively. Based on the clinical identification of kidney stone location, number, and size, the original study [12] gave a summary score for the disease severity for the patients with urolithiasis. In this study, an index of severity for the patients with kidney stones was defined by further classifying the summary score of the patient into different levels: 1 if score ≤ 4, 2 if score = 5 or 6, and 3 if score > 6.

### 2.5. Estimation of BMD Incorporating the Indirect Mediation Effect

The BMD given a predetermined benchmark response (BMR) is defined as the exposure level that satisfies the following relationship:

(7) $$P\left(BMD\right)-P_{0}=BMR,$$

where $P_{0}$ is the background response [25,26]. In this study, in addition to estimating BMD and BMDL directly from Equation (3), the mediation of MDA on the renal injury marker NAG based on Equation (6) was also considered. That is, we estimated the BMD by the DE of melamine exposure on NAG and the IE through the mediation of MDA on NAG. The BMD corresponding to a given BMR level, after some algebra, can be expressed as the solution of the following equation:

(8) $$\theta_{1}\left\{g\left(x\right)-g\left(x_{0}\right)\right\}+\theta_{2}\alpha_{1}\left\{log\left(x\right)-log\left(x_{0}\right)\right\}=\sigma \Psi ,$$

where Ψ $=\Phi^{-1}\left(1-P_{0}\right)-\Phi^{-1}\left(1-P_{0}-BMR\right)$, $\Phi^{-1}$ is the inverse of the standard normal density function, and $e^{x_{0}}-1$ is the EDI of melamine corresponding to the background response $P_{0}$ (Supplementary Materials, SM). To estimate $P_{0}$, a logistic model was employed for the dichotomous renal injury marker NAG of the participants for the estimated percentage of abnormalities at the 5% EDI of melamine. Following Lin and Tai-yi [27], the cutoff determined to be abnormal for NAG was >17.47 U/g Cr [14,15]. The background exposure level $x_{0}$ is the estimated EDI of melamine corresponding to $P_{0}$.

**Figure 1:**
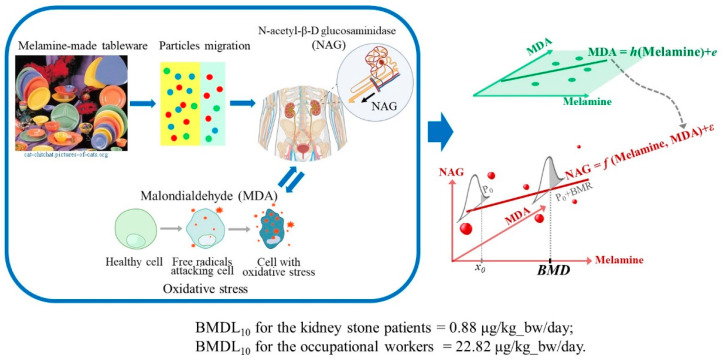
Schematic diagram of the BMD derivation incorporating the oxidative stress pathway between environmental exposure to melamine and the early renal injury marker NAG. The diagrams framed on the left illustrate the migration pathway of environmental exposure to melamine and ingestion into the human body, as well as the mechanistic pathway of the oxidative stress marker MDA between the melamine particles circulating within the body and the renal injury marker NAG. The upper-right figure shows the association between melamine exposure and the marker MDA. The lower-right figure explains the benchmark dose (BMD) calculation based on the regression model of melamine exposure and the response NAG in the presence of the composite effect of the mediator MDA.

We employed a Markov chain Monte Carlo (MCMC) simulation procedure using WinBUGS 1.4.3 (MRC Biostatistics Unit, Cambridge, UK) to obtain the corresponding model parameters. The BMD level was derived numerically. The corresponding BMDL level was designated as the value of the lower 95% of the BMD levels of the simulated samples (n = 10,000) upon convergence. We used Bayesian model averaging (BMA) with weights proportional to the reciprocal of the deviance information criterion (DIC) of the fitted model to obtain the final BMD and BMDL levels. Figure 1 illustrates the BMD estimation for the relationship between environmental exposure to melamine and the early renal injury marker NAG, considering the mediation pathway of the oxidative stress marker MDA.

## 3. Results

### 3.1. Baseline Characteristics and Urinary Measurements of the Two Study Cohorts

Table 1 shows the comparisons of the baseline characteristics and biomarker measurements of the spot-urine samples between the occupational workers and the patients with kidney stones. The occupational workers’ one-time collection of one-spot urine sample data was used for the comparisons. Both cohorts were predominantly men, with proportions over 70%. The workers were relatively younger compared to the patients with kidney stones (mean age: 44.8 vs. 54.7 years old) and had a lower BMI (mean 23.5 vs. 26.3 kg/m^2^) and percentage of smokers (13.8% vs. 33.8%), whereas the latter had higher proportions of comorbidity with diabetes mellitus (17.2%) and hypertension (35.6%).

Although more than 50% of the workers were from the control group, the overall mean urinary melamine concentration (209.46 μg/L) was much higher than that of the patients with kidney stones (12.25 μg/L). The mean EDI of the patients with kidney stones (1.10 μg/kg_bw/day) was approximately the same as that in the European countries (1.60 μg/kg_bw/day, Table 22) [1], whereas the mean EDI of melamine in occupational workers (27.27 μg/kg_bw/day) was much higher. Table S1 lists the percentiles of EDIs of the two study cohorts (SM). The maximum level of 178.7 μg/kg_bw/day was close to the recommended TDI of 200 μg/kg_bw/day by the WHO. The mean oxidative stress MDA level and the early renal injury marker NAG level of the occupational workers were also relatively higher than those of the patients with kidney stones (MDA: 2.06 vs. 1.16 μmol/L; NAG: 7.78 vs. 4.99 IU/L, respectively).

### 3.2. Associations between EDI of Melamine and Biomarker Measurements in Urine

**Figure 2:**
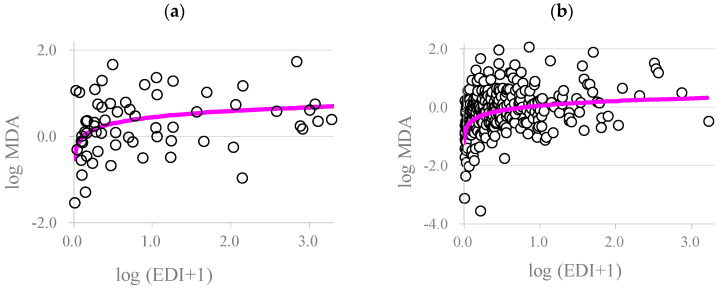
Scatter plot of the levels of the oxidative stress marker MDA in the (**a**) occupational workers and (**b**) patients with kidney stones and their EDIs of melamine. Both the MDA levels and the EDIs of melamine were logarithmically transformed. The circles are the observed MDA levels, and the pink line is the mean regression curve (Equation (4)).

**Figure 3:**
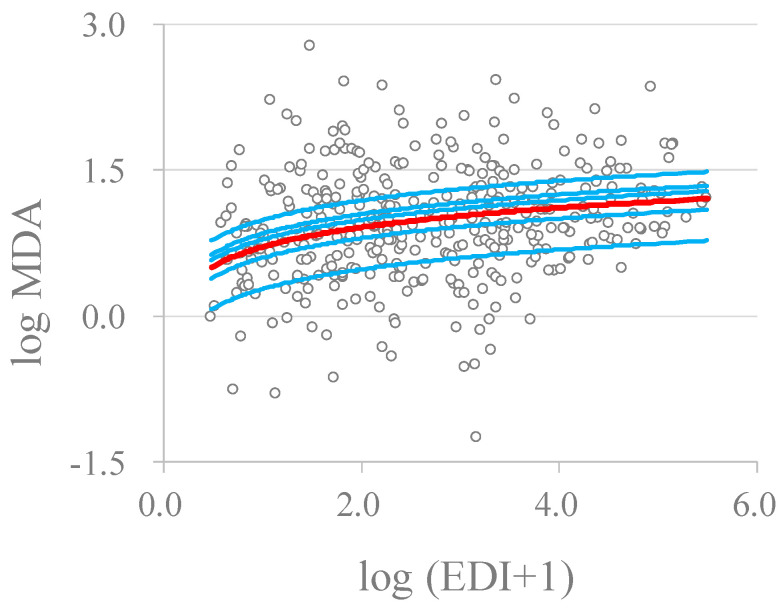
Scatter plot of the levels of the oxidative stress marker MDA in the melamine workers and their EDIs of melamine with repeated measurements. Both the MDA levels and the EDIs of melamine were logarithmically transformed. The circles are the observed MDA levels. The blue lines from the bottom are the 10%, 25%, 50%, 75%, and 90% of the 31 workers with repeated measurements of MDA and melamine in urine, and the red line is the overall mean regression curve (Equation (5)).

To serve as a mediator between melamine exposure and the health effect (NAG level), the regression term of the oxidative stress marker MDA level (logarithm-transformed) adjusted in the model must be significant (Equation (4). In addition, the association between the MDA level and the EDI of melamine must be significant (Equation (4)). Figure 2 shows the scatter plot of MDA (logarithm-transformed) and the EDI of melamine (logarithm-transformed), together with the fitted curve (Equation (4)) for the occupational workers (Figure 2a) and the patients with kidney stones (Figure 2b). The plot indicated a positive trend between the participants’ EDIs of melamine and the MDA levels, and the logarithmic model fit reasonably well, with the line going through the middle of the observations. Among the 38 workers of the melamine tableware factory, 31 individuals had repeated urine samples collected on 8 consecutive days. Figure 3 further supports the positive association between MDA and melamine EDI.

**Figure 4:**
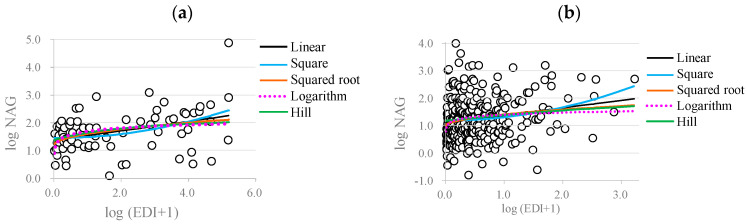
Scatter plot of the early renal injury marker NAG levels in the (**a**) occupational workers and (**b**) patients with kidney stones and their EDIs of melamine. Both the NAG levels and the EDIs of melamine were logarithm-transformed. The circles are the observed NAG levels. The black, blue, red, dotted pink, and green lines are the fitted statistical models with functional forms of linear, square, squared root, logarithm, and Hill models, respectively (Equation (6)).

Figure 4 displays the scatter plot of NAG and the EDI of melamine mediated by MDA and the fitted curves of various statistical models (Equation (6)). All the models had similar fitted curves across the central observations (the biomarker measurements and the EDI of melamine were logarithmically transformed). However, the fitted model curves of the squared root, logarithm, and Hill models were rather flat at the upper end, which would very likely yield a BMD level exceeding the maximum EDI of melamine.

The regression coefficients may be used to assess the magnitude of DE of melamine exposure on the renal injury marker NAG and the IE through the mediator (the oxidative stress marker MDA) on NAG. Table 2 summarizes the regression coefficient estimates between the subjects’ renal injury marker NAG level (logarithm-transformed) and the EDI of melamine $\beta_{1}$ (total effect) (Equation (1)), the oxidative stress MDA and the melamine EDI $\alpha_{1}$ (Equation (2)), and the NAG level and the EDI of melamine $\theta_{1}$ mediated by the MDA level $\theta_{2}$ (IE) (Equation (4)). For both cohorts, the total effect of the EDI of melamine on the early renal injury marker NAG was significant (*p* < 0.05) to highly significant (*p* < 0.001) for the different model fittings. The associations between MDA and the EDI of melamine were all highly significant. Similarly, the IE of MDA at the NAG level was also significant. However, after considering the mediation of MDA, the association between NAG and the EDI of melamine became insignificant except for the linear model fitting for the occupational workers, possibly due to the relatively small sample size (n = 80). The IE ($\beta_{1}-\theta_{1}$) of melamine exposure in the linear model fitting for the occupational workers and the patients with kidney stones were 0.09 (0.24−0.15) and 0.12 (0.30−0.18), respectively. That is, approximately 38% and 40% of the melamine daily exposure on the NAG level was mediated by the oxidative stress MDA for the workers and the patients, respectively. However, the IE interpretation may not be valid for the other nonlinear model fittings [24].

### 3.3. BMDLs without and with Considering the Mediation Effect of MDA

Table 3 lists the resulting BMDs and BMDLs, given a BMR of 0.10, for the occupational workers and the patients with kidney stones derived based on the total effect (without considering MDA) and the composite DE and the IE mediated by the oxidative stress marker MDA of melamine exposure on the renal injury marker NAG. Both the BMDs and BMDLs using the covariate-adjusted standardization method and the creatinine-adjusted EDIs of melamine are listed in the table. In addition to the adopted excretion fraction, *F_UE_* = 0.26, the outcomes using *F_UE_* = 0.90 are also listed for comparison.

Table 3a lists the BMDs and BMDLs of melamine exposure with model fittings of linear, square, squared root, logarithm, and Hill models ($g\left(x\right)=x,\;x^{2},\;x^{1/2},\;log\left(x\right),\;and\;Kx^{\alpha }/\left(1+Kx^{\alpha }\right)$) without considering the MDA mediation effect (Equation (1)). For the occupational workers, the BMD corresponding to the model fittings of squared root and logarithm were not shown due to exceeding the maximum EDI of melamine. Additionally, the BMDL of the logarithm model fitting also exceeded the maximum EDI and thus was omitted in the BMA. The BMDL_10_ of 18.09 μg/kg_bw/day after taking BMA was adopted as the exposure threshold for the workers. For the patients with kidney stones, the BMDL_10_ after taking BMA was 0.96 μg/kg_bw/day (BMD 1.98 μg/kg_bw/day), which was much smaller than that of the workers.

Table 3b summarizes the BMDs and BMDLs of various model fittings (Equation (4)) considering the composite DE and IE mediated by the oxidative stress marker MDA after melamine exposure. The BMD corresponding to the model fittings of the square root and Hill models for the workers is not shown due to exceeding the maximum EDI of melamine. The BMDL_10_ after taking BMA was 22.82 μg/kg_bw/day. For the patients with kidney stones, the BMDL_10_ was 0.88 μg/kg_bw/day (BMD 1.72 μg/kg_bw/day) based on BMA. In general, the BMDs and BMDLs adjusted for the mediator MDA remained approximately the same as those derived based on the total effect of melamine exposure without considering the mediator MDA.

In addition, Table 3 lists the BMDs and BMDLs with the EDIs estimated by using the creatinine-adjusted method (Equation (1)) and with the excretion fraction *F_UE_* = 0.90. In general, the creatinine-adjusted estimates had higher BMD and BMDL levels compared to those using the covariate-adjusted creatinine standardization method. The BMD and BMDL levels with the excretion fraction *F_UE_* = 0.90 were approximately one-third of those obtained by using *F_UE_* = 0.26. For reference, the BMDs and BMDLs given a BMR of 0.05 and 0.15 are listed in Table S2 and Table S3, respectively (SM). As expected, BMDL_05_ < BMDL_10_ < BMDL_15_ and the levels were approximately the same without and with adjustment by the mediator MDA for both the workers and the patients with kidney stones.

## 4. Discussion

In this study, by considering the mediation of the oxidative stress marker MDA, the exposure thresholds of melamine on the early renal injury marker NAG derived for patients with kidney stones and occupational workers were 0.88 μg/kg_bw/day and 22.82 μg/kg_bw/day, respectively. The BMDL_10_ levels without adjusting for MDA were similar (18.09 and 0.96 μg/kg_bw/day, respectively). However, the integration of DE and IE as mediators through the suggested mechanistic pathway between melamine exposure and early renal injury further strengthened the study outcomes. To the best of our knowledge, this is the first study reporting exposure thresholds considering suggested mechanisms based on human data.

Recently, adverse outcome pathway/pathway-based toxicology for the mode of action application to risk assessment (RA) has been advocated to address the controversy on the relevance of animal studies to human biology [28,29,30,31,32]. Many challenges remain, including the use of in vitro assays to identify perturbations of toxicity pathways at the molecular and cellular levels and other innovative quantitative approaches to understanding the shape of the dose–response curve [28,30,33,34]. Moreover, even among the most well-studied chemicals, very few of them had the type and quality of data needed for the new approach in RA [35]. The problem of limited toxicity data is further plagued by the complexity and challenges faced in the new approach method in dealing with uncertainties [31,33,36,37,38]. The proposed method may provide a reasonable and supportive approach based on observational data.

Oxidative stress is viewed as a pathological factor that leads to the initiation, development, and progression of most renal diseases [39,40]. Human kidneys are specifically sensitive to oxidative stress that is caused by the generation of pro-oxidants or reactive oxygen species exceeding endogenous antioxidant capacity [12]. Table 3 shows that melamine exposure was strongly associated with the oxidative stress marker MDA in both study cohorts. The mixed model fitting of the repeated measurements of urinary markers in the 31 workers from the melamine tableware factory further justified the association (Figure 3). Moreover, we showed that the proportion of the effect of melamine exposure on NAG levels mediated by MDA ranged from 38% to 40% in both study cohorts (linear model). The results supported the MOA of melamine exposure on early renal injury and the role of the oxidative stress marker MDA as a mediator in the pathway. A second oxidative stress marker, 8-oxo-2′-deoxyguanosine (8-OHdG), of the original dataset of Liu et al. [12] was also assessed for the role of mediation in the study. However, although the association between melamine exposure and the 8-OHdG level was highly significant, the association between 8-OHdG and NAG was insignificant. Thus, the corresponding BMD derivation incorporating 8-OHdG was ignored.

We did not derive the BMD of melamine exposure for the oxidative stress marker MDA as was performed in other studies on animals [41,42] and on omics profiles in a human cohort study [43]. Oxidative stress is a global marker of stress that is not necessarily specific to chemical exposure(s). Thus, it was deemed insufficient to be considered a health endpoint. Similarly, although epigenetic mediators may be associated with adverse health outcomes, the markers might function as IEs on the health outcomes rather than DEs in the context of dose–response modeling.

By comparing the baseline characteristics, the patients with kidney stones were shown to be an average of 10 years older than the workers and had higher comorbidity rates of diabetes and hypertension (Table 1). The patients with kidney stones also tended to have higher BMIs, higher smoking rates, and lower education levels. Together with the existing kidney problems of urolithiasis, these patients were much more vulnerable to melamine exposure. As a result, the corresponding exposure threshold level for the patients with kidney stones was much lower than that for the workers. Because of occupational exposure in the working environment, the mean urinary melamine concentration of 209.46 μg/L was more than one-order higher that of the patients (12.25 μg/L), even when averaged with that of the control group (42 workers from the steel company). This fact may also explain the higher exposure threshold for the workers. Nevertheless, the mean levels of MDA and NAG (2.06 μmol/L and 7.78 IU/L, respectively) in the workers were slightly higher than those in the patients with kidney stones (1.16 μmol/L and 4.99 IU/L, respectively), indicating latent oxidative stress and early kidney injuries resulting from occupational exposure to melamine.

The study outcomes suggested that the exposure threshold for melamine varied for different subpopulations depending on their health status and vulnerability. Table 4 summarizes the BMDL_10_ levels of melamine derived based on epidemiological studies and comparisons with the recommended TDIs based on an animal study in rats. Depending on the study subpopulation, the BMDL_10_ varied from 22.82 μg/kg_bw/day for occupational workers (healthy adults) to 0.74–2.03 μg/kg_bw/day for patients with early-stage CKD. The exposure threshold for patients with kidney stones was approximately the same as that for patients with early-stage CKD, whereas the exposure threshold for women who are pregnant (2.67 μg/kg_bw/day) was almost one order of magnitude lower than that for the workers, which was possibly due to physiological changes and thus increased vulnerability. However, all the exposure thresholds for melamine based on human data were one-third to two orders of magnitude lower than the TDI levels recommended by the WHO (EFSA) [44] and the US FDA [45] based on the same toxicological data.

The derived BMDL for the patients with kidney stones was lower than that of our previous study outcome of 4.89 μg/kg_bw/day [14]. The covariate-adjusted creatinine standardization method [23] was used for the EDI of melamine in this study instead of the creatinine-adjusted method [14]. The latter method tended to yield higher BMD and BMDL levels, as shown in Table 3. A higher background response $P_{0}$ (0.13) estimated in this study using a logistic model also led to a lower BMDL level, rather than based on sample proportion (0.06) and a creatinine-adjusted estimation of melamine EDI in a previous study [14]. In contrast, the occupational workers had a very low $P_{0}$ (0.002) of abnormal NAG levels due to being relatively healthy, young, and free from existing kidney problems. Because of the relatively small sample size, the modeling approach in this study should yield a more stable estimate of $P_{0}$.

There are some inherent limitations to the study. First, the BMDL levels for the occupational workers and the patients with kidney stones were derived based on cross-sectional measurements of melamine, MDA, and NAG in urine. The significant statistical associations between the EDI of melamine and MDA and NAG after adjusting for MDA clearly demonstrate the mediating role of MDA in the relationship between melamine exposure and early renal injury (Table 2). Thus, the adopted approach of BMD derivation, considering the mediation of MDA, fully supported the causal relationship. Moreover, the consistency between repeated measurements of MDA and melamine in urine for the melamine workers and that of the cross-sectional association further strengthened the causal association (Figure 2a and Figure 3). Second, the EDI of melamine was estimated based on a spot urine sample. High correlations between melamine concentrations in one-spot overnight urine samples and the previous 8- and 24-h urinary excretion of total melamine, as well as good correlations between the first- and second-day morning collections, have been shown in our previous studies [47,48]. Melamine concentrations in spot urine adjusted for creatinine can moderately predict urinary melamine levels over time, as suggested in a previous study [21]. However, random misclassification of exposure is still likely due to the fasting time effect. Third, this study did not consider serum uric acid or urinary cyanuric acid. Future studies are needed to collect measurements of cyanuric acid to control for potential confounding effects or to examine potential synergistic effects on markers of renal injury. Finally, we considered the marker NAG as the study endpoint. The marker may only suggest early renal injury rather than a pathological effect. Moreover, we considered only the oxidative stress marker MDA as the mechanistic pathway between melamine exposure and the NAG marker. There may have multiple pathways leading to renal injury. The present study, however, only serves an illustrative purpose for the proposed methodology. Further studies along this line are required.

## 5. Conclusions

In summary, the proposed method for deriving the exposure threshold of melamine by the composite DE and the IE mediated by the oxidative stress marker MDA provides a case study using human data with a suggested mechanistic pathway. The derived BMDL_10_ levels of melamine for occupational workers (22.82 μg/kg_bw/day) and patients with kidney stones (0.88 μg/kg_bw/day), consistent with our previous study outcomes, were subpopulation-dependent and up to two orders of magnitude lower than the current recommended TDI levels of 200 μg/kg_bw/day by the EFSA [1]. To prevent further clinical development, patients with existing kidney problems are advised to strictly refrain from food contact materials made of melamine.

## Figures and Tables

**Table 1:** Baseline characteristics of the occupational workers and the patients with kidney stones.

		Occupational Workers ^a^			Patients with Stones	
	Exposed	Non-Exposed		Overall		
			*p*-Value ^c^			*p*-Value ^c^
N (% of men)	38 (55.3)	42 (85.7)		80 (71.3)	309 (73.1)	
Education > 9 years (%)	15 (39.5)	41 (97.6)	<0.01	56 (70.0)	159 (51.5)	<0.01
Alcohol drinkers (%)	9 (23.7)	18 (42.9)	0.07	27 (33.8)	41 (13.3)	<0.01
Smokers (%)	9 (23.7)	2 (4.8)	0.01	11 (13.8)	104 (33.7)	<0.01
Diabetes mellitus (%)	2 (5.3)	2 (4.8)	0.92	4 (5.0)	53 (17.2)	<0.01
Hypertension (%)	3 (7.9)	10 (23.8)	0.05	13 (16.3)	110 (35.6)	<0.01
Dyslipidemia (%)	7 (18.4)	12 (28.6)	0.29	19 (23.8)	21 (6.8)	<0.01
Mean ± SD (median)						
Age (years)	42.37 ± 9.39 (44.00)	46.95 ± 10.63 (50.50)	0.01	44.8 ± 10.3 (49.0)	54.7 ± 14.8 (55.0)	<0.01
BMI (kg/m^2^)	23.51 ± 4.87 (22.95)	23.46 ± 2.87 (23.36)	0.43	23.5 ± 3.9 (23.2)	26.3 ± 4.1 (25.9)	<0.01
Urinary NAG concentration						
covariate-adjusted standardization (IU/L)	11.43 ± 20.84 (7.77)	4.49 ± 1.72 (4.22)	<0.01	7.78 ± 14.73 (5.16)	4.99 ± 5.67 (3.05)	<0.01
creatinine-adjusted (IU/mmol Cr)	1.10 ± 2.21 (0.74)	0.41 ± 0.16 (0.40)	<0.01	0.74 ± 1.56 (0.46)	1.35 ± 1.51 (0.87)	<0.01
Urinary MDA concentration						
covariate-adjusted standardization (μmol/L)	2.67 ± 3.56 (1.82)	1.50 ± 1.11 (1.11)	<0.01	2.06 ± 2.63 (1.43)	1.16 ± 1.03 (0.90)	<0.01
creatinine-adjusted (μmol/mmol Cr)	0.26 ± 0.38 (0.16)	0.14 ± 0.09 (0.10)	<0.01	0.19 ± 0.28 (0.13)	0.31 ± 0.28 (0.24)	<0.01
Urinary melamine concentration						
covariate-adjusted standardization (μg/L)	432.35 ± 451.26 (291.52)	7.80 ± 11.28 (3.40)	<0.01	209.46 ± 375.44 (16.01)	12.25 ± 25.42 (4.86)	<0.01
creatinine-adjusted (μg/mmol Cr)	41.21 ± 45.77 (23.96)	0.73 ± 1.04 (0.32)	<0.01	19.96 ± 37.36 (1.52)	3.24 ± 6.66 (1.26)	0.12
*EDI* of melamine (μg/kg_bw/day) ^b^						
covariate-adjusted standardization	43.96 ± 50.15 (23.36)	0.75 ± 1.16 (0.35)	<0.01	21.27 ± 40.62 (1.83)	1.10 ± 2.26 (0.45)	<0.01
creatinine-adjusted	29.76 ± 33.13 (17.52)	0.54 ± 0.71 (0.25)	<0.01	14.42 ± 27.02 (1.18)	2.40 ± 5.07 (0.96)	0.12

Notes: ^a^ Measurements in urine were taken on Friday morning. ^b^ Excretion fraction *F_UE_* = 0.26. ^c^ Group differences for categorical data were tested by using the chi-square test, and Kruskal–Wallis test for continuous variables.

**Table 2:** Coefficient estimates of the regression models for the statistical associations between the early renal injury marker NAG and melamine EDI ($\beta_{1}$: total effect) (Equation [3]), the oxidative stress marker MDA and the EDI of melamine ($\alpha_{1}$) (Equation [4]), and NAG and melamine EDI ($\theta_{1}$) with mediation of MDA ($\theta_{2}$) (Equation [6]). The adjusted covariables for the covariate-adjusted creatinine standardization for ${\hat{c}}_{r}$ included age, sex, and BMI; and the creatinine-adjusted method was adjusted by the participant’s creatine level in the voided spot urine.

		Occupational Workers ^a^ (n = 80)				Patients with Kidney Stones ^b^ (n = 309)			
Adjustment of Urinary Markers	Model	$\beta_{1}$	$\alpha_{1}$	$\theta_{1}$	$\theta_{2}$	$\beta_{1}$	$\alpha_{1}$	$\theta_{1}$	$\theta_{2}$
Covariate-adjusted creatinine standardization	linear	0.24 ***	0.22 ***	0.15 *	0.34 **	0.30 **	0.23 ***	0.18 *	0.19 **
	square	0.04 *	0.23 ***	0.03 ^b^	0.25 *	0.09 **	0.23 ***	0.09 *	0.20 **
	squared root	0.46 ***	0.21 ***	0.22	0.42 ***	0.47 **	0.23 ***	0.28 *	0.20 **
	logarithm	0.13 *	0.21 ***	0.02	0.48 ***	0.11 **	0.22 ***	0.07	0.15 *
	Hill	3.15 ***	0.21 ***	1.11	0.21 ***	2.17 **	0.22 ***	0.78 *	0.15 *
Creatinine-adjusted	linear	0.19 ***	0.21 ***	0.12 *	0.41 ***	0.18 **	0.26 ***	0.10	0.21 ***
	square	0.05 ^b^	0.21 ***	0.03 ^b^	0.42 ***	0.06 **	0.26 ***	0.04 *	0.21 ***
	squared root	0.43 **	0.21 ***	0.21	0.45 ***	0.34 **	0.26 ***	0.18 ^b^	0.21 ***
	logarithm	0.16 ***	0.21 ***	0.07	0.46 ***	0.12 **	0.26 ***	0.06	0.20 ***
	Hill	3.04 ***	0.21 ***	0.81	0.58 ***	1.38 *	0.26 ***	0.63	0.28 ***

Notes: ^a^ Covariables adjusted in the model included age, sex, BMI, cigarette smoking, and working factory. ^b^ Covariables adjusted in the model included age, sex, BMI, cigarette smoking, and kidney stone index. *: *p* < 0.05; **: *p* < 0.01; ***: *p* < 0.001.

**Table 3:** Benchmark dose (BMD) and the corresponding 95% lower bound (BMDL) of melamine exposure on the renal injury marker NAG (a) without and (b) with mediation of the oxidative stress marker MDA for the occupational workers and the patients with kidney stones. The Bayesian model averaging (BMA) was obtained by the weighted average of the fitted models (linear, square, square root, logarithm, and Hill) with the weight reciprocal to the DIC of the fitted model. The excretion fraction *F_UE_* was the fraction of the average daily intake (EDI) of melamine excreted in urine. The adjusted covariables for the covariate-adjusted creatinine standardization for ${\hat{c}}_{r}$ included age, sex, and BMI; and the creatinine-adjusted method was adjusted by the participant’s creatine level in the voided spot urine.

(a)								
			**Occupational Workers ^a^ (n = 80)**			**Patients with Kidney Stones ^b^ (n = 309)**		
***F_UE_***	**Adjustment for Urinary Markers**	**Model**	**BMD ^c^**	**BMDL ^c^**	**DIC**	**BMD**	**BMDL**	**DIC**
0.26	Covariate-adjusted creatinine standardization	Linear	40.39	10.79	411.7	1.62	0.80	1491.6
		Square	75.32	33.12	408.6	5.15	2.72	1497.1
		Square root	―	18.05	426.2	0.80	0.33	1478.9
		Log	―	―	433.8	0.49	0.15	1472.6
		Hill	46.21	10.10	420.6	1.86	0.80	1487.4
		BMA		18.09		1.98	0.96	
								
	Creatinine-adjusted	Linear	―	44.83	48.5	4.45	2.04	686.2
		Square	103.17	50.11	46.8	7.85	4.75	687.5
		Square root	―	36.83	50.1	2.36	0.90	686.1
		Log	―	61.61	52.5	0.96	0.32	685.1
		Hill	―	33.88	74.1	6.39	1.88	705.0
		BMA		46.15		4.39	1.98	
								
0.9	Covariate-adjusted creatinine standardization	Linear	11.48	4.61	407.6	0.81	0.43	1497.0
		Square	31.19	14.66	409.4	2.63	1.40	1491.4
		Square root	34.37	3.95	420.6	2.70	2.11	1485.6
		Log	―	―	432.9	0.16	0.05	1474.0
		Hill	14.60	4.60	416.3	1.11	0.51	1494.7
		BMA		6.98		1.48	0.90	
								
	Creatinine-adjusted	Linear	―	13.70	48.4	1.68	0.91	685.9
		Square	25.08	15.20	45.2	3.05	2.07	687.6
		Square root	―	11.59	49.3	0.93	0.39	685.9
		Log	―	18.17	51.9	0.34	0.11	685.0
		Hill	―	10.93	71.7	2.51	1.08	703.6
		BMA		14.09		1.70	0.91	
(b)								
			**Occupational Workers ^a^ (n = 80)**			**Patients with Kidney Stones ^b^ (n = 309)**		
***F_UE_***	**Adjustment for Urinary Markers**	**Model**	**BMD ^c^**	**BMDL**	**DIC**	**BMD**	**BMDL**	**DIC**
0.26	Covariate-adjusted creatinine standardization	Linear	111.21	13.74	628.2	1.52	0.87	1963.8
		Square	79.67	30.51	622.1	3.68	2.20	1970.2
		Square root	―	24.80	638.8	0.89	0.40	1954.5
		Log	131.83	12.46	642.8	1.39	0.34	1960.1
		Hill	―	32.46	636.6	1.15	0.59	1961.2
		BMA		22.82		1.72	0.88	
								
	Creatinine-adjusted	Linear	―	27.40	319.6	2.63	1.07	440.4
		Square	107.44	35.10	318.6	4.05	1.89	440.0
		Square root	―	24.95	320.7	1.89	0.65	440.9
		Log	28.58	5.13	322.3	1.45	0.65	441.2
		Hill	―	47.73	326.9	2.09	0.80	447.3
		BMA		28.02		2.42	1.01	
0.9	Covariate-adjusted creatinine standardization	Linear	16.17	4.49	622.3	0.70	0.42	1977.6
		Square	30.28	12.92	623.6	1.72	1.00	1974.8
		Square root	―	4.77	635.1	0.32	0.16	1967.0
		Log	49.19	6.08	643.5	0.72	0.10	1968.0
		Hill	―	6.95	632.5	0.59	0.30	1976.2
		BMA		7.06		0.81	0.40	
								
	Creatinine-adjusted	Linear	―	9.82	319.7	0.88	0.42	435.7
		Square	―	21.46	319.0	1.57	0.77	435.7
		Square root	―	4.16	320.9	0.56	0.24	436.5
		Log	5.09	1.17	322.3	0.79	0.32	437.2
		Hill	―	5.31	327.5	0.69	0.33	441.7
		BMA		8.42		0.90	0.42	

Notes: ^a^ Covariables adjusted in the model included age, sex, BMI, cigarette smoking, and working factory. ^b^ Covariables adjusted in the model included age, sex, BMI, cigarette smoking, and kidney stone index. ^c^ The “―” means the estimated BMD (BMDL) exceeded the maximum EDI level.

**Table 4:** Summary of melamine exposure thresholds (TDI/BMDL) derived based on animal and epidemiological studies.

Study Subjects	Health Endpoint	Background Response P_0_	Point of Departure (μg/kg_bw/day)	Uncertainty Factor	TDI/BMDL (μg/kg_bw/day)	Study
Animal study						
Rats (male)	bladder stones	0	35,000 (BMDL)	200	200	[44]
Rats (male)	bladder stones	―	63,000 (NOAEL)	1000	63	[45]
Rats (male)	bladder stones	0	8090 (BMDL_05_)	1000	8.09	[46] ^a^
Epidemiological studies						
Children (≤5 years old)	nephrolithiasis	0.001		―	8–30	[7]
Patients with urolithiasis	renal injury marker NAG	0.06	8.27 (BMD)	―	4.89 ^c^	[14]
Patients with early-stage chronic kidney disease	• time to doubling of serum creatinine levels • eGFR decline over time	0.05–0.25	0.80–4.04 (BMD)	―	0.74–2.03	[13]
Women who are pregnant (third trimester)	renal injury marker NAG	0.08–0.22	5.44 (BMD)	―	2.67 (1.46 ^b^)	[15]
Occupational workers	renal injury marker NAG	0.002	― ^c^	―	22.82 (18.09 ^d^)	This study
Patients with urolithiasis	renal injury marker NAG	0.13	1.72 (1.98 ^d^) (BMD)	―	0.88 (0.96 ^d^)	This study

Notes: ^a^ The highest dosage group of the study was excluded. ^b^ Considering co-exposure to DEHP up to the 90th percentile. ^c^ The derived BMD exceeded the maximum EDI of melamine. ^d^ Without a mediation effect of the oxidative stress marker MDA.

## Data Availability

The raw data would be made available by the second author (Chia-Chu Liu) upon request.

## Supplementary Materials

The following supporting information can be downloaded at: https://www.mdpi.com/article/10.3390/toxics12080584/s1, Table S1. Percentiles of the EDI (μg/kg_bw/day) of melamine of t; Table S2. Benchmark dose (BMD) and the corresponding 95% lower bound (BMDL), given BMR = 0.05 and 0.15, of melamine exposure on the renal injury marker NAG (a) without; and (b) with mediation of the oxidative stress marker MDA for the occupational workers (N = 80); Table S3. Benchmark dose (BMD) and the corresponding 95% lower bound (BMDL), given BMR = 0.05 and 0.15, of melamine exposure on the renal injury marker NAG (a) without; and (b) with mediation of the oxidative stress marker MDA for the stone patients (N = 309).
