# Supplementary material for: Benchmark Dose of Melamine Exposure for a Renal Injury Marker Mediated by Oxidative Stress: Examples in Patients with Urolithiasis and Occupational Workers
Source: Toxics. 2024 Aug 11;12(8):584. doi: 10.3390/toxics12080584 (PMC11359403; doi:10.3390/toxics12080584)
Supplement: Supplementary file 1 [file toxics-12-00584-s001.zip › toxics-3110950-supplementary.pdf]

Supplementary Materials

Derivations of Eq. (8)

Following Eqs. (4) & (6), the mean μ of $\log(NAG_i)$ can be written as

$$\mu\{\log(NAG_i)|x\} = \theta_0 + \theta_1 g(x) + \theta_2 log(MDA|x) + \boldsymbol{\gamma}'_3\boldsymbol{Z}$$
$$= \theta_0 + \theta_1 g(x) + \theta_2\{\alpha_0 + \alpha_1 log(x_i) + \boldsymbol{\gamma}'_2\boldsymbol{Z}\} + \boldsymbol{\gamma}'_3\boldsymbol{Z}.$$

Thus, following the definition of BMD (Eq. [7]),

$$\frac{\mu\{\log(NAG_i)|x\} - \mu\{\log(NAG_i)|x_0\}}{\sigma} = \Phi^{-1}(1-P_0) - \Phi^{-1}(1-P_0-BMR) = \Psi,$$

where Φ^{-1} is the inverse of the standard normal density function, $e^{x_0} - 1$ is the EDI of melamine corresponding to the background response P_0, and $\sigma^2 = \sigma_m^2 + \sigma_N^2$. The Eq. (8) follows after some algebra.

Table S1. Percentiles of the EDI (μg/kg_bw/day) of melamine of the participants.

	N	Adjustment method	F_{UE}	5th	25th	75th	90th	Maximum
Occupational workers	80	covariate-adjusted-standardization based	0.26	0.080	0.334	20.818	68.130	178.665
		creatinine-adjusted based		0.055	0.233	16.385	50.726	116.411
		covariate-adjusted-standardization based	0.9	0.023	0.097	6.014	19.682	51.614
		creatinine-adjusted based		0.016	0.067	4.733	14.654	33.630
Stone Patients	309	covariate-adjusted-standardization based	0.26	0.025	0.160	1.100	2.502	24.043
		creatinine-adjusted based		0.056	0.359	2.475	5.017	54.353
		covariate-adjusted-standardization based	0.9	0.007	0.046	0.318	0.723	6.946
		creatinine-adjusted based		0.016	0.104	0.715	1.449	15.702

Table S2. Benchmark dose (BMD) and the corresponding 95% lower bound (BMDL), given BMR = 0.05 and 0.15, of melamine exposure on the renal injury marker NAG (a) without; and (b) with mediation of the oxidative stress marker MDA for the occupational workers (N = 80).

(a)

F_{UE}	Adjustment of urinary markers	Model[a]	BMD=0.05		BMD=0.15		
			BMD[b]	BMDL	BMD[b]	BMDL[b]	DIC
0.26	Covariate-adjusted	Linear	17.5	5.96	70.66	15.89	411.7
		Square	44.88	21.53	103.58	43.08	408.6
		Square root	60.93	6.09	—	40.89	426.2
		Log	—	22.90	—	—	433.8
		Hill	14.46	4.93	115.16	16.69	420.6
		BMA	—	12.26	—	29.13	
	Creatinine-adjusted	Linear	74.11	18.91	—	79.96	48.5
		Square	59.34	31.20	—	67.10	46.8
		Square root	113.32	9.57	—	104.64	50.1
		Log	—	3.85	—	—	52.5
		Hill	55.74	12.07	—	68.69	74.1
		BMA	—	15.70	—	80.83	
0.9	Covariate-adjusted creatinine standardization	Linear	6.18	2.85	17.19	6.26	407.6
		Square	20.39	10.34	40.39	18.13	409.4
		Square root	8.61	1.82	—	6.78	420.6
		Log	—	3.86	—	—	432.9
		Hill	6.29	2.59	27.19	6.74	416.3
		BMA	—	4.31	—	9.50	
	Creatinine-adjusted	Linear	18.69	7.14		21.00	48.4
		Square	16.78	10.68	32.05	18.85	45.2
		Square root	25.23	3.99	—	25.71	49.3
		Log	—	1.54	—	—	51.9

		Model	BMD	BMDL	BMD	BMDL	DIC
		Hill	13.07	5.13	—	18.69	71.7
		BMA	—	5.88	—	21.20	

(b)

F_{UE}	Adjustment of urinary markers	Model[a]	BMR = 0.05		BMR = 0.15		
			BMD	BMDL	BMD	BMDL	DIC
0.26	Covariate-adjusted	Linear	28.97	6.17	—	23.06	628.2
		Square	40.28	17.00	121.77	42.83	622.1
		Square root	—	6.25	—	76.51	638.8
		Log	19.41	4.58	—	29.82	642.8
		Hill	—	8.40	—	99.52	636.6
		BMA	—	8.52	—	54.24	
	Creatinine-adjusted	Linear	53.39	9.04	—	57.34	319.6
		Square	46.29	16.40	—	53.94	318.6
		Square root	—	5.34	—	90.49	320.7
		Log	7.29	2.33	104.14	9.85	322.3
		Hill	—	7.99	—	—	326.9
		BMA	—	8.24	—	52.96	
0.9	Covariate-adjusted creatinine standardization	Linear	6.71	2.43	29.05	6.56	622.3
		Square	17.05	7.87	42.51	17.23	623.6
		Square root	15.33	1.78	—	9.84	635.1
		Log	10.13	2.57	—	12.64	643.5
		Hill	14.96	2.83	—	13.34	632.5
		BMA	—	3.51	—	11.92	
	Creatinine-adjusted	Linear	20.58	2.33	—	26.43	319.7
		Square	—	4.43	—	—	319.0

Square root	9.72	1.16	—	11.33	320.9
Log	2.12	0.68	10.84	1.75	322.3
Hill	16.67	1.25	—	17.76	327.5
BMA	—	1.98	—	14.33	

[a] Covariables adjusted in the model included age, sex, BMI, cigarette smoking, and working factory.
[b] The "—" means the estimated BMD (BMDL) exceeded the maximum EDI level.

Table S3. Benchmark dose (BMD) and the corresponding 95% lower bound (BMDL), given BMR = 0.05 and 0.15, of melamine exposure on the renal injury marker NAG (a) without; and (b) with mediation of the oxidative stress marker MDA for the stone patients (N = 309).

(a)

F_{UE}	Adjustment of urinary markers	Model[a]	BMD=0.05 BMD	BMD=0.05 BMDL	BMD=0.15 BMD	BMD=0.15 BMDL	DIC
0.26	Covariate-adjusted	Linear	0.71	0.4	2.83	1.27	1491.6
		Square	2.82	1.64	7.60	3.74	1497.1
		Square root	0.27	0.14	1.78	0.60	1478.9
		Log	0.12	0.07	2.41	0.33	1472.6
		Hill	0.73	0.38	3.73	1.32	1487.4
		BMA	0.93	0.52	3.66	1.45	
	Creatinine-adjusted	Linear	1.58	0.88	9.54	3.66	686.2
		Square	4.00	2.64	12.21	6.93	687.5
		Square root	0.65	0.34	7.20	1.89	686.1
		Log	0.24	0.14	5.37	0.70	685.1
		Hill	1.73	0.79	20.37	3.00	705.0
		BMA	1.64	0.96	10.89	3.24	
0.9	Covariate-adjusted creatinine standardization	Linear	0.39	0.22	1.30	0.64	1497.0
		Square	1.59	0.91	3.61	1.86	1491.4
		Square root	2.03	1.87	4.05	2.47	1485.6
		Log	0.04	0.02	0.67	0.11	1474.0
		Hill	0.47	0.24	1.99	0.80	1494.7
		BMA	0.90	0.65	2.32	1.18	
	Creatinine-adjusted	Linear	0.72	0.43	2.96	1.46	685.9
		Square	1.81	1.29	4.24	2.77	687.6
		Square root	0.28	0.15	2.25	0.77	685.9
		Log	0.08	0.05	1.53	0.25	685.0

		Hill	0.94	0.51	5.42	1.81	703.6
		BMA	0.76	0.49	3.27	1.41	

(b)

F_{UE}	Adjustment of urinary markers	Model	BMR = 0.1 BMD	BMR = 0.1 BMDL	BMR = 0.15 BMD	BMR = 0.15 BMDL	DIC
0.26	Covariate-adjusted	Linear	0.56	0.33	2.91	1.52	1963.8
		Square	1.49	0.64	6.20	3.50	1970.2
		Square root	0.26	0.16	2.30	0.79	1954.5
		Log	0.52	0.12	4.28	0.83	1960.1
		Hill	0.37	0.21	2.54	1.08	1961.2
		BMA	0.64	0.29	3.64	1.54	
	Creatinine-adjusted	Linear	0.63	0.34	7.75	2.39	440.4
		Square	1.07	0.43	8.30	4.10	440.0
		Square root	0.42	0.23	8.20	1.51	440.9
		Log	0.70	0.36	3.13	1.10	441.2
		Hill	0.45	0.26	9.27	1.91	447.3
		BMA	0.65	0.32	7.33	2.20	
0.9	Covariate-adjusted creatinine standardization	Linear	0.27	0.14	1.25	0.70	1977.6
		Square	0.68	0.21	2.75	1.56	1974.8
		Square root	0.10	0.06	0.72	0.30	1967.0
		Log	0.29	0.03	1.86	0.28	1968.0
		Hill	0.19	0.08	1.21	0.57	1976.2
		BMA	0.31	0.10	1.56	0.68	
	Creatinine-adjusted	Linear	0.25	0.13	1.92	0.86	435.7
		Square	0.44	0.15	2.75	1.59	435.7

Square root	0.14	0.09	1.59	0.51	436.5
Log	0.41	0.17	1.54	0.54	437.2
Hill	0.18	0.10	1.81	0.74	441.7
BMA	0.28	0.13	1.92	0.85	

[a] Covariables adjusted in the model included age, sex, BMI, cigarette smoking, and stone index.
